# A Cross-Sectional Study to Assess the Healthcare-Seeking Behaviour of Tribal Communities in a District of Maharashtra

**DOI:** 10.7759/cureus.85687

**Published:** 2025-06-10

**Authors:** Dinesh Asokan, Nita Gate, Rakesh Waghmare, Anjali Mall, Geeta Pardeshi

**Affiliations:** 1 Community Medicine, Grant Government Medical College and Sir JJ Group of Hospitals, Mumbai, IND

**Keywords:** healthcare access, healthcare-seeking behaviour, informal healthcare services, tribal communities, vulnerable populations

## Abstract

Introduction

Tribal populations in India face longstanding barriers to accessing formal healthcare due to economic, geographic, and cultural constraints. This study assessed the healthcare-seeking behaviour of tribal households in Palghar district, Maharashtra, and examined associated determinants.

Methods

A community-based cross-sectional study was conducted from August 2023 to March 2024 using multistage cluster random sampling in eight tribal villages located within a 25 km radius of the district hospital. A total of 80 households were selected, and 306 individuals were enumerated. Of these, 84 individuals (27.5%) who reported illness in the past three months were included in the analysis. Data were collected using a pretested structured questionnaire and analysed using R software (R Foundation for Statistical Computing, Vienna, Austria). Chi-square tests were applied to assess associations between healthcare-seeking behaviour and independent variables.

Results

Only 25 (29.8%) of the ill individuals sought formal healthcare, while 29 (34.5%) accessed informal providers, and 30 (35.7%) took no action. Among all variables analysed, only perceived severity of illness was significantly associated with formal healthcare utilization. Formal care was accessed by 13 of 14 (92.9%) individuals who perceived their illness as severe, compared to 11 of 40 (27.5%) with moderate and five of 30 (16.7%) with mild perception. No significant associations were found with age, gender, education, number of symptoms, or timing of illness.

Conclusion

The study highlights low formal healthcare utilization and a strong influence of perceived illness severity on care-seeking behaviour. Continued reliance on spiritual healers and non-action reflects persistent cultural and structural barriers. Interventions should include culturally sensitive health promotion, expansion of nearby healthcare services, and financial support mechanisms. Further qualitative research is needed to explore contextual factors influencing healthcare choices in tribal communities.

## Introduction

Healthcare-seeking behaviour refers to the actions individuals take in response to perceived health issues, including decisions about when and where to seek care. This behaviour significantly influences health outcomes. Timely and appropriate healthcare utilization can lead to early diagnosis and effective treatment, thereby reducing morbidity and mortality [1]. Conversely, delays or avoidance in seeking care may result in disease progression and increased healthcare costs. Understanding these behaviours is essential for developing public health strategies that promote equitable access to healthcare services and improve population health [2].

Tribal populations represent some of the most marginalized groups, often facing unique challenges in accessing healthcare. In India, tribal communities constitute approximately 8.6% of the total population, equating to over 104 million individuals [3]. These communities frequently reside in remote areas and maintain distinct cultural practices that influence their health-seeking behaviours. Limited exposure to formal healthcare systems further exacerbates the challenges these populations face [4]. Addressing the healthcare needs of tribal populations is crucial for reducing health disparities and achieving inclusive health development [5].

The Palghar district in Maharashtra is notable for its substantial tribal population, with 37.39% identified as Scheduled Tribes according to the 2011 Census. Predominant tribes in this region include the Malhar Koli, Warli, and Katkari, many of whom are engaged in daily wage labour and agriculture [6]. Despite the district's proximity to urban centres, these communities encounter significant barriers to healthcare access, including geographical isolation and economic constraints. Many tribal households in the region continue to rely on traditional healing practices and informal healthcare providers, reflecting broader patterns observed in tribal settings across India [7].

The study objectives are to describe the healthcare-seeking behaviour of tribal communities in Palghar district and to examine illness-related characteristics associated with the type of healthcare accessed. These descriptive findings aim to inform future research and guide the development of culturally sensitive strategies to enhance formal healthcare utilization among tribal populations.

## Materials and methods

Study design and setting

This community-based cross-sectional study was conducted from August 2023 to March 2024 in Palghar district, Maharashtra. Palghar is a tribal-dominated district in western India, where Scheduled Tribes constitute 37.39% of the total population, according to Census 2011 [8]. The study focused on tribal villages located within a 25 km radius of the District Hospital, Palghar.

Sampling methodology

A multi-stage random sampling technique was employed to ensure the geographical representativeness of tribal communities.

**Figure 1:**
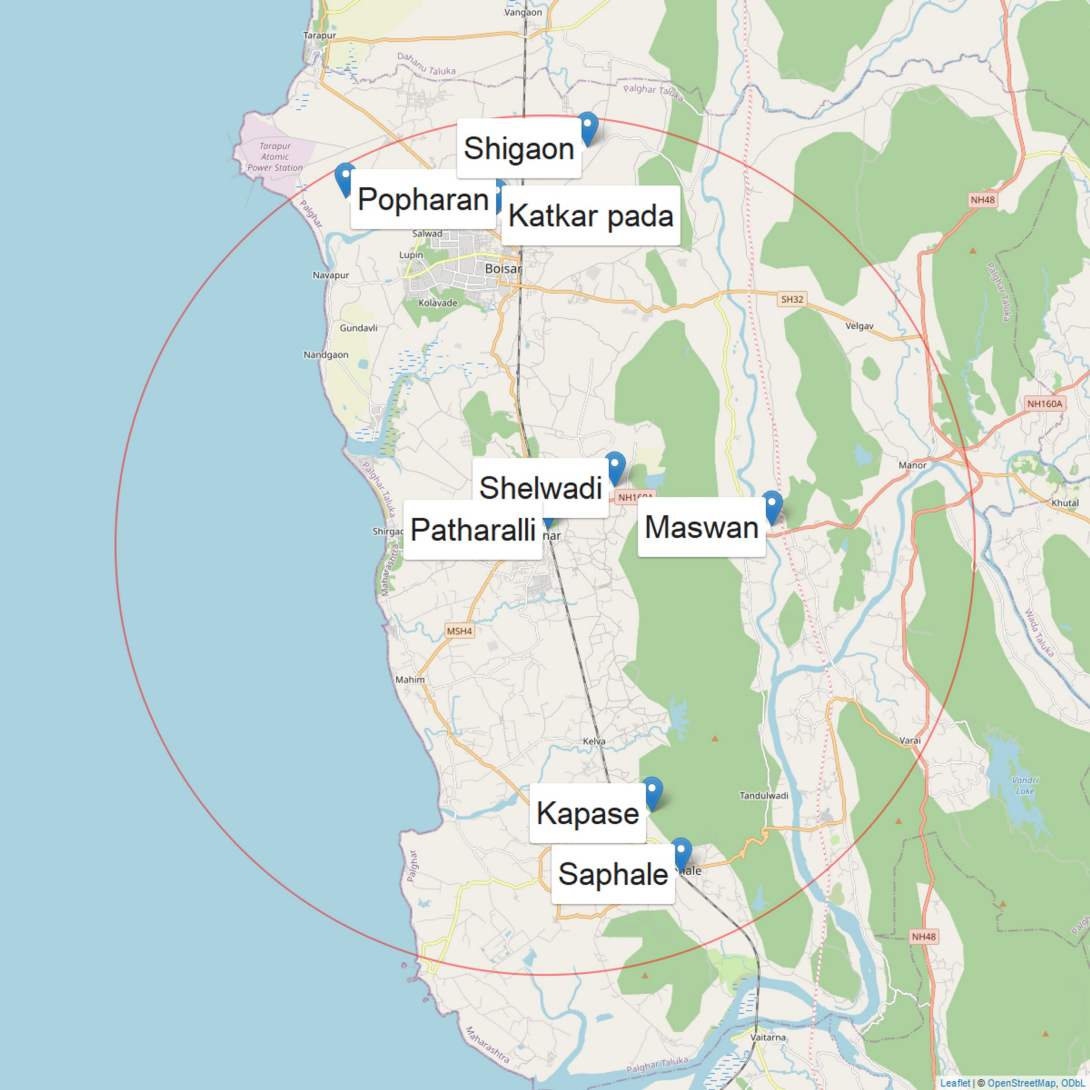
Geographic representation of village selection randomly using geographic information system. Generated by the first author (Dinesh Asokan) using R software version 4.5.2.

In the first stage, a list of all tribal villages located within a 25 km radius of the District Hospital was prepared based on local administrative records and validated through the health department. Using QGIS software (QGIS Development Team, Gossau, Switzerland), these villages were geocoded and stratified into four quadrants, north, east, south, and west, with the hospital at the centre. From each quadrant, two villages were randomly selected using the random number generator within QGIS, resulting in a total of eight study villages (Figure 1).

In the second stage, a complete household listing was conducted in each selected village in collaboration with Accredited Social Health Activists (ASHAs) and anganwadi workers. Using simple random sampling, 10 households per village were selected from the household list using a random number table. This yielded a total of 80 tribal households (8 villages × 10 households).

Each selected household was visited, and all permanent members were enumerated. From this pool, individuals who had experienced any self-reported illness in the preceding three months were eligible for inclusion in the healthcare-seeking behaviour analysis. If a selected household did not have any member who experienced illness in the past three months, it was retained in the enumeration but excluded from the healthcare-seeking behaviour analysis. In households with more than one eligible individual, all such individuals were included for analysis to capture intra-household variation in healthcare-seeking behaviour.

Sample size calculation

The required sample size was calculated using the Statulator online sample size calculator for estimating a single proportion. Assuming that 90% of the tribal population would exhibit some form of healthcare-seeking behaviour [9], with a 5% absolute precision, 95% confidence level, and a design effect (DEFF) of 2 to account for cluster sampling, the estimated required sample size was 277 individuals. With an average of three to seven members per household, the sampling of 80 households was expected to yield sufficient coverage. The final enumerated population was 306 individuals, among whom 84 individuals had experienced illness in the three-month recall period and were analysed for healthcare-seeking behaviour.

Study participants

All permanent residents of the selected households were enumerated. Individuals who had experienced any acute or chronic illness in the past three months were included in the analysis of healthcare-seeking behaviour. Individuals temporarily residing elsewhere or with incomplete data were excluded.

Study tool and data collection

A pretested, structured questionnaire was used for data collection. The questionnaire was developed in English, translated into Marathi, and then back-translated to ensure linguistic accuracy. It was validated for content by three public health experts and pretested in a tribal village not included in the final study. Minor modifications were made based on pilot feedback.

The questionnaire covered socio-demographic profile, illness characteristics (type, severity, timing, and number of symptoms), first action taken following illness onset, healthcare-seeking behaviour (formal vs. informal or no action), and reasons for not accessing formal care [10].

Formal care was defined as services accessed at government health facilities or private clinics/hospitals. Informal care included spiritual healers, traditional practitioners, home remedies, and self-treatment.

Data were collected through face-to-face interviews conducted in Marathi by trained investigators under supervision. Responses were recorded using paper-based forms and later digitized. Perceived severity of illness was self-reported by participants and categorized into mild, moderate, or severe based on their own understanding, without any clinical judgment or interviewer categorization. All included responses were complete, and no missing data imputation was required during the analysis.

Ethical considerations

The study was approved by the Institutional Ethics Committee, Grant Government Medical College & Sir J.J. Group of Hospitals, Mumbai (Approval No: IEC/Pharm/RP/187/May/2024; dated: 15/05/2024). Written informed consent was obtained from all adult participants. For minors, assent was obtained along with parental consent. Participant anonymity and data confidentiality were strictly maintained.

Data analysis

Data were cleaned and entered in Microsoft Excel (Microsoft Corporation, Redmond, WA) and analysed using R software, version 4.3.2 (R Foundation for Statistical Computing, Vienna, Austria). Descriptive statistics were used to summarize socio-demographic characteristics, illness patterns, and healthcare-seeking behaviour. Categorical variables were expressed as frequencies and percentages (n, %). Subgroup analysis was performed for illness-related variables, including number of symptoms, timing of occurrence, and self-perceived severity of illness, using the chi-square/Fisher's exact test. A p-value < 0.05 was considered statistically significant.

## Results

The study comprised 80 households, predominantly belonging to the Malhar Koli community, with 70 (87.5%) households. Hindu Dubali households accounted for six (7.5%), while Warli Koli households represented four (5.0%). Across these households, details of 306 individuals were recorded, of whom 84 (27.5%) reported symptoms in the past three months. Febrile illnesses were the most common complaints, with fever reported in 36 (42.85%) of the cases.

Table 1 presents the socio-demographic characteristics of the 84 (100%) household members who reported illness in the past three months. A higher proportion was of females (45, 53.57%), and the majority were married (45, 53.57%). Most belonged to the working-age group, with 24 (28.57%) aged 41-60 years and 19 (22.62%) aged 19-25 years. Educational attainment was low, with 34 (40.47%) being illiterate. Daily wage earning was the predominant occupation at 64 (76.19%), reflecting the economic vulnerability of the study population.

**Table 1:** Socio-demographic details of household members who perceived illness in the past three months (n = 84).

Variable	Category	Frequency	Percentage
Gender	Male	39	46.43
	Female	45	53.57
Marital status	Single	28	33.33
	Married	45	53.57
	Widow	11	13.10
Age category	Less than 18	17	20.24
	19–25	19	22.62
	26–40	15	17.86
	41–60	24	28.57
	Above 60	4	4.76
Education	Illiterate	34	40.47
	Primary school	23	27.38
	Middle school	13	15.48
	High school	10	11.90
	Intermediate/diploma	3	3.57
Occupation	Daily wage earner	64	76.19
	Agriculture	19	22.62
	Small business	1	1.19

Table 2 presents the first action taken by 84 (100%) household members for perceived illness. A total of 30 (35.71%) took no action, including 25 (29.76%) who acknowledged the need but did not act. Informal care was preferred by 29 (34.52%), mainly through spiritual healers (17, 20.24%) and home remedies (9, 10.71%). Only 25 (29.77%) sought formal healthcare, with 15 (17.86%) visiting private providers and 10 (11.91%) accessing public health services. These findings indicate a predominant reliance on informal and passive responses over formal medical care.

**Table 2:** First action taken for perceived illness by household members (N = 84).

Variable	Category	Frequency	Percentage
No action	Did not need any action	5	5.95
	Perceived need but took no action	25	29.76
Informal care	Medical shops/pharmacists	3	3.57
	Spiritual healers	17	20.24
	Self-treatment/home remedies	9	10.71
Formal care	Public health centres	10	11.91
	Private clinics/hospitals	15	17.86

Table 3 explores the association between illness-related factors and the type of healthcare accessed by 84 (100%) respondents. The number of symptoms and timing of illness did not show statistically significant associations with healthcare-seeking behaviour (p = 0.145 and p = 0.756, respectively). However, perceived severity of illness was significantly associated with healthcare access (χ² = 26.17, p < 0.001).

**Table 3:** Association between illness-related characteristics and type of healthcare accessed (n = 84).

Variables	Categories	Formal care (n)	Informal/no action (n)	Chi-square value	p-value
Number of symptoms	One	19	44	2.12	0.145
	Two	10	11		
Occurrence time	Before a month	7	15	0.1	0.756
	Within a month	22	40		
Perceived severity of Illness	Mild problem	5	25	26.17	<0.001
	Moderate problem	11	29		
	Severe problem	13	1		

Table 4 outlines the reasons given by 56 (100%) individuals for consulting informal service providers instead of formal healthcare. The most frequently reported reasons were lack of money (20, 23.80%) and distance to health facilities (16, 19.04%). Other notable reasons included less belief in modern medicine (13, 15.47%), perceived less severity of symptoms (3, 3.57%), non-availability of doctors (2, 2.38%), and poor quality of services (2, 2.38%). These findings highlight the combined influence of financial, geographical, and cultural factors in shaping healthcare-seeking behaviour.

**Table 4:** Reasons for consulting informal service providers instead of formal (N = 56).

Reason	Frequency	Percentage
Lack of money	20	23.80
Distance of health facility	16	19.04
Less belief in modern medicine	13	15.47
Less severity of symptoms	3	3.57
Non-availability of doctors	2	2.38
Poor quality of service	2	2.38

## Discussion

The findings of this study shed light on the socio-demographic and healthcare-seeking behaviour patterns of tribal households in the Palghar district of Maharashtra, revealing persistent barriers to formal healthcare access and continued reliance on informal systems. The predominance of the Malhar Koli tribe (87.50%) reflects the localized tribal composition of the study area, consistent with Sarmah and Dutta (2019), who noted that health-seeking behaviour is often shaped by geographically clustered social networks within tribal populations [11]. Economic vulnerability was evident, with 76.19% of the ill individuals engaged in daily wage labour. Similar observations have been made by Mishra et al. (2015), who highlighted the role of poverty in pushing tribal communities toward informal healthcare pathways [12].

Febrile illnesses were the most frequently reported health issue, with fever affecting nearly half of the total individuals. This is consistent with national and regional data highlighting the high burden of febrile illnesses among tribal populations due to factors such as poor sanitation, vector-borne diseases, and limited preventive services [13]. Although 26.19% of participants delayed seeking care, this delay was not significantly associated with the type of healthcare accessed in our analysis, indicating that factors other than timing may more strongly influence the decision-making process. This contrasts with prior reports that linked delayed care primarily to geographical and financial limitations [9].

A key and novel finding of this study was the statistically significant association between perceived severity of illness and healthcare-seeking behaviour. Among those who rated their illness as severe, 92.9% accessed formal healthcare services substantially higher than those with moderate (27.5%) or mild (16.7%) self-assessments. This association (χ² = 26.17, p < 0.001) aligns with the illness-perception model, suggesting that perceived threat level drives more structured healthcare engagement. This complements findings by Boro and Saikia (2020), who observed that tribal populations often delay care until conditions are perceived as critical [14].

Informal healthcare remained a dominant choice, with 34.56% opting for spiritual healers or self-treatment. Many participants preferred spiritual healers due to strong cultural beliefs and traditional health practices. Common methods included ritual-based healing, the use of indigenous herbs, and prayer ceremonies. These practices are part of the community’s health-seeking behaviour and reflect high cultural acceptability and trust in local healing systems. Such reliance often delays contact with formal healthcare services, highlighting the need for culturally sensitive health promotion strategies. This aligns with the findings of Sudhinaraset et al. (2013), who attributed reliance on informal providers to affordability and cultural familiarity [15]. Overall, formal healthcare utilization was low (29.77%), consistent with Kanungo et al. (2015), who cited both cultural and systemic constraints in tribal settings [16].

Barriers to formal healthcare access in this study were multifactorial, encompassing both structural and cultural factors. The most commonly reported obstacles were financial constraints (20, 23.80%) and distance to health facilities (16, 19.04%), underscoring the role of economic hardship and geographic inaccessibility. These findings align with those of Ghosal et al. (2023), who highlighted structural deficits, including lack of transport, unaffordable care, and remote facility locations, as key deterrents in tribal health access [10].

Cultural beliefs also played a substantial role. Mistrust in modern medicine and preference for traditional healing practices were cited by 13 (15.47%) individuals, reflecting the deep-rooted reliance on familiar, community-embedded systems of care. This is consistent with earlier studies that found spiritual healers and home-based remedies were often preferred over biomedical services due to cultural compatibility and longstanding trust [17,18].

Despite a moderate overall healthcare utilization rate of 52.38%, a significant portion of those experiencing mild or moderate symptoms did not seek formal care. This indicates that perceptions of illness severity, coupled with access-related and cultural barriers, continue to hinder timely engagement with formal health systems. These findings echo the work of Chandwani and Pandor (2015), who emphasized that limited trust, low perceived quality of care, and insufficient community-level awareness are persistent challenges in improving healthcare access among tribal populations [19].

Strengths and limitations

This study employed a multi-stage random sampling strategy to enhance geographic representation across tribal villages. The use of a structured and pretested questionnaire ensured consistency in data collection. A three-month recall period was chosen to minimize recall bias, which is a common limitation in behavioural surveys. Ethical protocols were rigorously followed, and appropriate statistical tests were applied with clearly reported p-values, contributing to methodological transparency and validity.

Villages were selected within a 25 km radius of the district hospital, which may limit the generalizability of findings to more remote tribal areas. Only 10 households were surveyed per village, which may not fully capture variations in healthcare-seeking behaviour. The severity of illness was self-reported, introducing subjectivity. Cultural interpretations of illness and providers were not explored in depth. The modest sample size of 84 individuals with illness also limits subgroup analysis. Lastly, the study did not adopt a formal behavioural theory framework, although the examined variables align conceptually with established models like Andersen’s behavioural model.

## Conclusions

This study highlights significant gaps in the utilization of formal healthcare services among tribal households in Palghar district. A substantial proportion of individuals with recent illness either took no action or relied on informal providers, most commonly spiritual healers. Perceived severity of illness emerged as a strong determinant of formal care-seeking, while those with milder symptoms were less likely to engage with the health system. Structural barriers, such as financial hardship and geographic inaccessibility, were frequently reported, alongside perceptual barriers like mistrust in modern medicine and preference for culturally familiar practices.

While the study observed associations between healthcare-seeking behaviour and factors such as symptom burden and perceived severity, these findings should not be interpreted as causal due to the cross-sectional design and small subgroup sizes. Further qualitative research is needed to explore the cultural beliefs and contextual barriers underlying these patterns. To improve healthcare utilization in tribal regions, public health interventions must be both structurally enabling and culturally sensitive. Strategies should include expanding service reach to remote areas, reducing financial barriers, and fostering trust in the public health system through consistent service delivery and community engagement. Emphasis on early care-seeking for even moderate symptoms may help shift patterns away from informal or delayed care.
